# Carrier-free multi-components self-delivery nanocomplex for tumor synergistic therapy

**DOI:** 10.1016/j.ijpx.2025.100443

**Published:** 2025-11-12

**Authors:** Pengkai Ma, Ziqi Jing, Xue Wang, Xiaoya Liu, Zirui Tan, Yujie Zhang, Zhijun Wang

**Affiliations:** a School of Chinese Materia Medica, Beijing University of Chinese Medicine, Beijing 100029, China; b Division of interventional radiology, Department of Geriatric Medicine, The Second Medical Center & National Clinical Research Center for Geriatric Diseases, Chinese PLA General Hospital, Beijing 100039, China; c Department of Intervenional Radiology, Chinese PLA General Hospital, Beijing 100853, China

**Keywords:** Nanomedicine, Synergistic therapy, Metal-phenolic network, Nanocrystal

## Abstract

Due to the intricate nature of tumors, developing multidrug delivery system to enhance synergistic therapy for tumors is urgently needed. Herein, we present a carrier-free nanocomplex (DHT NC@SF MPN), consisting of dihydrotanshinone-1 nanocrystals (DHT NC) combined with silybin-ferric metal-phenolic network coatings (SF MPN), for multidrug self-delivery and chemo/chemodynamic synergistic cancer therapy. The generated nanocomplex (DHT NC@SF MPN) was completely composed of three therapeutic ingredients: DHT (56.08 %), Sil (41.88 %), and Fe(III) (2.01 %) without incorporation of any nano-materials. It displayed spherical core-shell structure with particle size 262 nm and acid responding drug release. The nanocomplex could be efficiently uptaken by HGC-27 tumor cells through the lipid raft/caveolin mediated pathway. It exhibited stronger tumor cell proliferation inhibition, migration inhibition, cell apoptosis and ferroptosis compared with free drugs. On tumor bearing mice, it showed comparable anti-tumor efficacy with the commercial paclitaxel liposome while systemic toxicity was negligible. Therefore, the facile nanocarrier-free multidrug self-delivery nanoplatform shed light on developing advanced nanomedicine for tumor synergistic therapy.

## Graphical abstract

**Figure:**
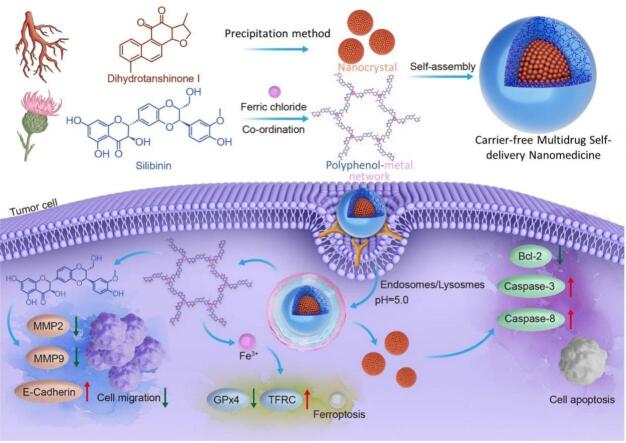

## 1 Introduction

Despite major advances in therapy, cancer continues to be a leading cause of mortality (Bray et al., 2021). Given the complexity of the disease, a single chemotherapeutic drug or therapy modality is often insufficient to achieve satisfactory treatment outcome. Combination therapy, which integrates multi-drugs and modalities, often produces enhanced curative effects due to synergistic interactions. This approach has become a routine treatment strategy in clinical practice (Jiang et al., 2022; Yang et al., 2022). Herbal medicine is an important part of combination therapy, it works by its various active components that acting on multi-targets. *Salvia miltiorrhiza* and *Silybum marianum* are widely used herbal medicine. Ongoing research has revealed that their active components, dihydrotanshinone Ι (DHT) and silybin (Sil), exhibit significant potential in suppressing tumor growth, metastasis, and recurrence (Jia et al., 2024; Verdura et al., 2024; Yassin et al., 2022). However, akin to many conventional chemotherapeutic drugs, low solubility, poor bioavailability, and side effects have hindered their clinical utilization. Meanwhile, the codelivery of several anticarcinogens which possess distinct mechanisms and active sites was capable of effectually achieving superior therapeutic results.

The past decades have witnessed the emergence of carrier-free nanomedicine. Compared with the traditional nanomedicine, the carrier-free nanomedicine has significant advantages in the following aspects: since it is solely composed of several active agents without or with minimum application of nonactive excipients, so it has obviously inherent advantage in enhancing drug loading and avoiding undesired side effects caused by excipients; it can be fabricated through one-step approach, which is more simple, scalable, and cost-effective (Fu et al., 2022; Shim et al., 2022). For instance, drug nanocrystals (NC) are generally recognized as a representative carrier-free nanomedicine. They consist only of active ingredients and essential stabilizers. Thus, drug nanocrystals have been an atractive strategy in addressing the unfavorable physicochemical and pharmacokinetic properties of poorly soluble anticancer drugs, such as paclitaxel, camptochecin, quercetin, *etc.*.

Metal-polyphenol network (MPN) is a supramolecular porous membrane self-assembled by the coordination interactions between phenolic hydroxyl groups and metal ions (Ping et al., 2015; Zheng et al., 2017). It is well known that polyphenols are a large class of active substances in natural plants, many of them have been approved by the FDA for utilization in food and medicine (Patra et al., 2021; Rajendran et al., 2022; Sajadimajd et al., 2020). And the metal ions can also play an important role in bioimaging or anti-tumor (Chen et al., 2022a; Chen et al., 2022b; Hu et al., 2022). So, sometimes it can serve as a special carrier-free nanomedicine. For instance, tea polyphenol (Shan et al., 2019), curcumin (Greish et al., 2018), shikonin (Feng et al., 2022) can coordinate with Fe^3+^, Pt^2+^, or Cu^2+^ to form MPNs through a simple one-pot method, and the coordinate bond maintain stable under physiological condition, while particularly dissociate in acid or reductive tumor microenvironment. Moreover, thanks to the universal adhesive property of the MPN membrane, it is also suitable for serving as functional coatings *via* polymerization on various inorganic/organic substrates, such as mesoporous silica nanoparticle, gold nanorod, and dendrimers, *etc.* This further broaden its application in therapeutic delivery and bioimaging to boost tumor theranostics (Fan et al., 2017; Wang et al., 2022; Xu et al., 2022; Zhang et al., 2020).

**Fig. 1:**
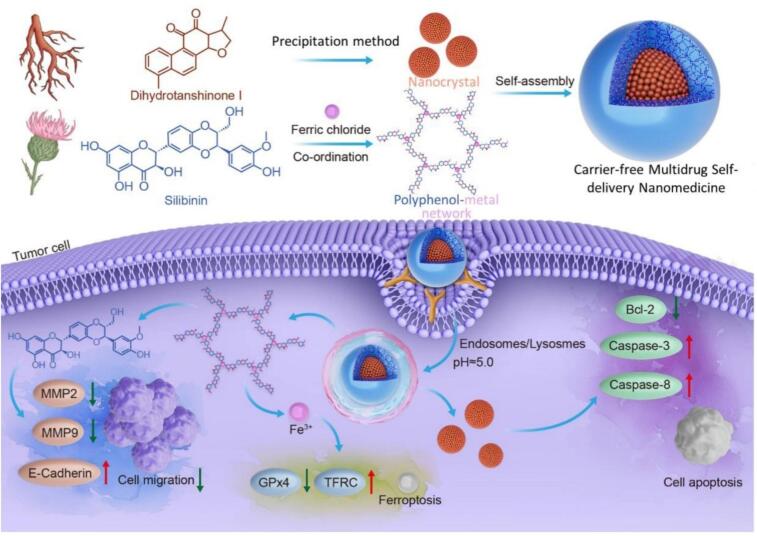
Formation of DHT NC@SF MPN nanocomplex for synergistic therapy of tumors.

Inspired by the advantages of combination therapy and carrier-free nanomedicine, we intend to employ Sil as the polyphenol coordinating with ferric to form silybin-ferric metal-phenolic network (SF MPN) and further coated on the surface of dihydrotanshinone-1 nanocrystal (DHT NC), thus constructing a carrier-free multi-drug self-delivery nanomedicine (DHT NC@SF MPN). As shown in Fig. 1, the DHT NC@SF MPN firstly adhere to tumor after intra-tumoral injection due to its bio-adhesive property and then the SF MPN decomposes into DI NC, Sil and Fe(*III*)due to its acidic responsibility. DHT and Sil tend to block the cell cycle of tumor cells, produce apoptotic bodies to suppress tumor cell proliferation and promote tumor cell apoptosis. Meanwhile, Fe(III) triggers ferroptosis of tumor cells. Finally, these three active substances jointly kill tumor through a multiple pathways action mode.

## 2 Materials and methods

### 2.1 Materials

Dihydrotanshinone I (DHT, 98 %) was purchased from Chengdu herbsubstance Co. Ltd. (Chengdu, China). Silybin (Sil, 99 %) and anhydrous ferric chloride (FeCl_3_, 99 %) were purchased from Shanghai DB Biological Science and Technology Co. Ltd. (Shanghai, China). IR808 (1-(5-carboxypentyl)-2-[2-[3-[[1-(5-carboxypentyl)-1,3-dihydro-3,3-dimethyl-2H-indol-2-ylidene]ethylidene]-2-chloro-1-cyclohexen-1-yl]ethenyl]-3,3-dimethyl-3H-indolium bromide, 99 %) was purchased from Sigma (St. Louis, MO, USA). Roswell Park Memorial Institute-1640 (RPMI-1640), fetal bovine serum (FBS), and other cell culture reagents were purchased from Gibco (Grand I., Empire State, USA). MitoTracker Green, Lyso-Tracker Green DND-99, ER-Tracker Green, Golgi-Tracker Green, and Hoechst 33342 were purchased from Aladdin Co. Ltd. (Beijing, China). Chlorpromazine hydrochloride, nystatin, methyl cyclodextrin, amiloride hydrochloride, cytochalasin D were purchased from Beyotime Biotechnology (Shanghai, China). Glutathione (GSH), reactive oxygen species (ROS), and Fe^2+^ assay kits were purchased fron Nanjing Jiangong Co. Ltd. (Nanjing, China). Antibodies were obtained from Abcam (Shanghai) Co. Ltd. (Shanghai, China). Paclitaxel liposome injection (Lps-P) was purchased from Nanjing Luye Pharmaceutical Co. Ltd. (Nanjing, China). BALB/c nude mice were supplied by Vital River Laboratory Animal Technology Co., Ltd. (Beijing, China).

### 2.2 DHT NC preparation

DHT NC was prepared using the precipitation method as described elsewhere with minor modification (Morakul et al., 2014). Briefly, 5 mL DHT acetone solution (1 mg·mL^−1^) was dropped into 25 mL poly (vinyl pyrrolidone) K30 (PVP K30) aqueous solution (1 mg·mL^−1^) and vigorously stirred for 30 min at room temperature. High-pressure homogenization (Noozle nano lab type, China) was applied to reduce the particle size. After diluting with 10 times volume of water, acetone was removed by rotary evaporation at 37 °C under reduced pressure. DHT NC was obtained following freeze drying. The influence of PVP K30 dose, stirring speed, pressure and cycle number of high-pressure homogenizations on the particle size was studied by single factor and orthogonal experiment. In order to track cellular uptake and intracellular distribution, fluorescent IR808-doped DHT NC (DHT/IR808 NC) was also prepared. IR808 was mixed with DHT at molar ratio of 1:100, and following process was the same with DHT NC.

### 2.3 DHT NC@SF MPN preparation

DHT NC suspension (800 μL, 1 mg·mL^−1^) was added with Sil DMSO solution (300 μL, 18 mmol·L^−1^) and FeCl_3_ solution (100 μL, 18 mmol·L^−1^). 4 mL tris buffer (20 mmol·L^−1^, pH = 8.5) was added to the mixture, and whisk the mixture vigorously for 2 min. The mixture was centrifuged at 15000 rpm for 10 min to collect the precipitates and washed with water for five times. The resulting product DHT NC@SF MPN was obtained following freeze drying. The drug loading, encapsulation efficiency, particle size and acid sensitivity were selected as the indexes for optimizing the preparation process of the SF MPN. The DHT NC@SF MPN prepared from different ratios of Sil to Fe(III) was placed in buffer solution with different pH for 1 h, and the DHT and Sil content was determined to evaluate the acidity sensitivity of SF MPN.

### 2.4 Characterization

**Chemical structure** X-ray diffraction (XRD, X'Pert Pro MPD, Netherlands) and fourier transform infrared (FTIR, Nicolet iS10, USA) spectroscopy were employed to confirm the molecular structure and crystal phase of DHT NC and DHT NC@SF MPN. XRD spectroscopy was scanned at 3.0°/min from 3.0° to 60°. 2 mg DHT NC or DHT NC@SF MPN powder was mixed with 200 mg KBr powder and pressed into a transparent KBr pellet, the FTIR spectra was recorded in the 0–4000 cm^−1^ range at a resolution of 2 cm^−1^.

**Size distribution, zeta potential and morphology** DHT NC or DHT NC@SF MPN was dispersed in ultrapure water at a concentration of 10 mg·mL^−1^ and a dynamic light scattering (DLS) nano particle size analyzer (Nicomp 380, Santa Barbara, CA) was used to determine the size distribution and zeta potential. Further, scanning electron microscopy (SEM, Sigma 300 ZEISS, Germany) and transmission electron microscopy (TEM, JEOL JEM-2100, Japan) were used to observe the morphology of DHT NC and DHT NC@SF MPN.

**Drug loading and encapsulation efficiency** To determine the content of DHT, Sil and Fe(*III*)in DHT NC@SF MPN, the DHT NC@SF MPN was dissolved in phosphate buffer solution (PBS pH 4.5) to degrade the coordination bond between Sil and Fe^3+^. Then the DHT and Sil content was determined using an ultrahigh performance liquid chromatography (UPLC, Agilent 1290, USA) (supplementary Fig.S1A&S1B). In addition, the iron ion content was determined by the o-phenanthroline UV–visible spectrophotometry method (UV–Vis, TU-1901, Beijing, China) (supplementary Fig.S1C).

The following formulas were adopted to calculate the loading and encapsulation efficiency (EE):

$$\mathrm{DL}\left(\%\right)=\frac{\text{amount of drug in}\;\mathrm{DHT}\;\mathrm{NC}@\mathrm{SF}\;\mathrm{MPN}}{\text{weight of}\;\mathrm{DHT}\;\mathrm{NC}@\mathrm{SF}\;\mathrm{MPN}}\times 100\%$$

$$\mathrm{EE}\left(\%\right)=\frac{\text{amount of drug in}\;\mathrm{DHT}\;\mathrm{NC}@\mathrm{SF}\;\mathrm{MPN}\;}{\text{amount of drug added}}\times 100\%$$

**Stability** DHT NC or DHT NC@SF MPN (9 mg each) were dispersed in 24 mL PBS, and equal portions (3 mL) were taken daily to measure the particle size.

***In vitro* drug release** Dialysis method was used to assess the *in vitro* drug release behavior as described previously. Briefly, DHT and DHT NC@SF MPN suspensions (1 mL each) were packed in dialysis bags and immersed in 50 mL PBS (pH 6.0, 7.4 and 8.8) containing 2 % Tween 80. Dialysis bags were shaken at 100 rpm at 37 °C. At predefined time point, 1 mL release medium was pipetted out and replaced with fresh media at the same volume. The concentration of DHT and Sil was determined using the UPLC method. The cumulative rates of drug release were calculated with the following formula, where Cc was the corrected concentration, Cd was the measured concentration, F(t) was the calculated cumulative release rate, and Wt was the drug content in the nanocomplex.

$$\mathrm{Cc}=\mathrm{Cd}+1/50\sum_{i=1}^{n=1}\mathrm{Cd}$$

$$F\left(t\right)=\frac{\mathrm{Cc}\times 50}{\mathrm{Wt}}\times 100\%$$

### 2.5 Cytotoxicity

The cytotoxicity was evaluated on HGC-27 cells through the 3-(4,5-dimethylthiazol-2-yl)-2,5-diphenyltetrazolium bromide (MTT) test method. HGC-27 cells were seeded into 96-well plates at a density of 1 × 10^5^ cells/well. After reaching logarithmic growth phase, drug solution was added with a series of concentration (0.5–25.0 μmol·L^−1^, calculated as DHT) and co-incubated with cells for 24 h or 48 h. Then, culture medium was discarded and MTT solution (20 μL, 5 mg·mL^−1^) was added, diluted with culture medium to 200 μL and co-incubated with cells for 4 h. Afterwards, culture medium was replaced with DMSO and incubated for 10 min to dissolve the forming formazan crystal. Finally, a micro-plate reader (SPECTRO star Nano, Germany) was used to record the absorbance at 490 nm of each well.

### 2.6 Apoptosis

YF488-Annexin-V/PI double staining method was performed to label apoptotic cells and a flow cytometer (Cyto FLEX, Suzhou, China) was used to determine the apoptosis rate (Dong et al., 2022). HGC-27 cells were seeded in 6-well plates at a density of 4 × 10^5^ cells/well. After reaching logarithmic growth phase, drug solution (5.0 μmol·L^−1^, calculated as DHT) were added and co-incubated with cells for 24 h. Cells were then digested with trypsin, washed with pre-cooled saline for 3 times, and treated with 5 μL YF488-AnnexinV/PI for 30 min at 4 °C in darkness. The fluorescent emission light intensity at 530/665 nm was detected by the flow cytometer with excitation light at 488 nm.

### 2.7 Migration of cells

The effect on tumor cell migration was investigated by cell wound scratch assay (Grada et al., 2017). When HGC-27 cells seeded in 6-well plates reached logarithmic growth phase, a 200 μL pipette tip was used to carve the bottom of the 6-well plate to produce a straight incision. After discarding the floating cells, drug solutions (5.0 μmol·L^−1^) were added and co-incubated with cells for 12 h. A microscope was used to observe the closure of scratches wound at 0 h and 12 h. The following formula was used to calculate cell migration rate. S_0_ and St represented the scratch area at 0 h and 12 h, respectively.

$$\text{Cell migration}\;\left(\%\right)=\frac{S0-\mathrm{St}}{S0}\times 100\%$$

### 2.8 Ferroptosis

Ferroptosis was evaluated by determining levels of reactive oxygen species (ROS), and Fe^2+^. HGC-27 cells were seeded in 6-well plates at a density of 4 × 10^5^ cells/well. After 24 h, drug solutions (5.0 μmol·L^−1^) were added and co-incubated with cells for 24 h. Cells were trypsinizated, collected and lysed by sonication. DCFH-DA and FerroOrange staining were used to determine the content of ROS and Fe^2+^, respectively. They were observed by an inverted fluorescence microscope (Eclipse Ti2—U Nikon, Tokyo, Japan). Fluorescence intensity was analyzed by the Image J software.

### 2.9 Cellular uptake and intracellular distribution

IR808 fluorescence-doped DHT/IR808 NC and DHT/IR808 NC@SF MPN were prepared and used to investigate the cellular uptake. HGC-27 cells were seeded in 35 mm glass bottom dishes. After reaching logarithmic growth phase, drug solutions (4.0 μmol·L^−1^, calculated by IR808) was added and co-incubated with cells for 4 h. The cells were washed with PBS for three times. Then, CLSM was used to observe the fluorescence of cells. In addition, to investigate the effect of drug concentration and incubation time on cellular uptake, the DHT/IR808 NC@SF MPN with different concentrations (1.0, 2.0, 4.0 μmol·L^−1^) was co-incubated with cells for different time (1.0, 2.0, 4.0 h). To illustrate the cellular distribution of DHT/IR808 NC@SF MPN after entry into cells, cells were further treated with various organelle probes for another 30 min, such as MitoTracker Green (20 nmol·L^−1^), Lyso-Tracker Green DND-99 (75 nmol·L^−1^), ER-Tracker Green (200 nmol·L^−1^), Golgi-Tracker Green (5 μmol·L^−1^) and Hoechst 33342 (1 mg·mL^−1^). Fluorescent images captured by the confocal laser scanning microscope were overlapped to locate the sub-cellular distribution of the DHT/IR808 NC@SF MPN, and the Image J software was used to calculate Pearson's R value for co-localization analysis.

### 2.10 Cellular uptake pathways

HGC 27 cells were seeded in 6-well plates. When reaching logarithmic growth phase, the proteins that mediating different endocytosis pathways were blocked by pre-treating with inhibitors for 30 min, such as chlorpromazine hydrochloride (30 μmol·L^−1^, clathrin), nystatin (30 μmol·L^−1^, caveolin), methyl cyclodextrin (10 μmol·L^−1^, lipid raft/caveolin), amiloride hydrochloride (40 μmol·L^−1^, macropinocytosis) and cytochalasin D (5 μmol·L^−1^, globular actin) (Yan et al., 2019). Then, DHT NC@SF MPN (10 μmol·L^−1^) was added and co-incubated with cells for 4 h. After washing the cells with PBS to remove free DHT NC@SF MPN, the cells were trypsinzed, re-suspended and lysed with SDS after a 4 h incubation at 37 °C. UPLC was used to determine intracellular DHT concentration.

### 2.11 Evaluation of *in vivo* pharmacodynamics

BALB/c nude mice (male, 20 ± 2 g) were inoculated with HGC 27 cells (2 × 10^8^ cells/mL) in the right armpit. When the tumor volume reached 80 ∼ 100 mm^3^, 48 nude mice were randomly divided into 8 groups (*n* = 6): model group, saline group, DHT group, DHT NC group, DHT + Sil group, DHT + Sil + Fe(III) group, DHT NC@SF MPN group and paclitaxel liposome (Lps-P, Lipusu®) group. Mice were intra-tumoral administered with different drugs at a dosage of 5 mg/cm^3^ (calculated as DHT or paclitaxel), once every other day, and the administration cycle was 14 days. During the whole experiment, the tumor volume was measured manually with a digital vernier caliper, and the tumor volume (mm^3^) = length×width^2^ × 0.5. During the treatment, body weight of mice was recorded. At the end of the experiment, blood was collected by the eyeball removal method, and serum was obtained after the blood was centrifugated at 3500 rpm for 15 min. Aspartate aminotransferase (AST), alanine aminotransferase (ALT), creatinine (CRE), and blood urea nitrogen (BUN) were analyzed by the assay kits. Tumor and normal tissues (heart, liver, spleen, lung, and kidney) were removed, weighed, immobilized with 4 % paraformaldehyde, and sectioned for hematoxylin-eosin (H&E) staining.

### 2.12 Terminal deoxynucleotidyl transferase-mediated 20′-deoxyuridine-50′-triphosphate-biotin nick end labeling (TUNEL) assay

The tumor tissues were immersed in paraffin for 15 min, treated with formaldehyde, and then sectioned. The terminal deoxynucleotidyl transferase-mediated 20′-deoxyuridine-50′-triphosphate-biotin nick end labeling (TUNEL) dye and proteinase K working solution were co-incubated with the paraffin sections after they had been washed in dimethylbenzene and gradient ethanol. A fluorescent microscope (Olympus BX51, Japan) was used to take pictures of each section.

### 2.13 Western blot

Tumor tissues (0.2 g) were cut into pieces and lysed with 1 mL RIPA lysate buffer. After centrifugation at 12000 *g* for 20 min at 4 °C, the supernatant was collected and the total protein content was determined using a BCA protein quantification kit. The protein concentration was diluted to 10 mg/mL, and western blotting was used to determine the expression of B-cell lymphoma-2 (Bcl-2), caspase-3, caspase-8, E-cadherin, matrix metalloproteinase 2 (MMP2), glutathione peroxidase 4 (GPX4) and transferrin receptor (TFRC), which were related to tumor apoptosis, metastasis and ferroptosis.

### 2.14 Statistical analysis

Data were shown as mean ± standard deviation (*n* = 6). SPSS 22.0 statistical software was used to analyze the statistical significance between the groups. The Student's *t*-test was used to compare data between two groups, and one-way analysis of variance (ANOVA) was used to compare data between multiple groups. When *p* < 0.05 was considered a statistically significant difference.

## 3 Results and discussion

### 3.1 DHT NC@SF MPN preparation

**Fig. 2:**
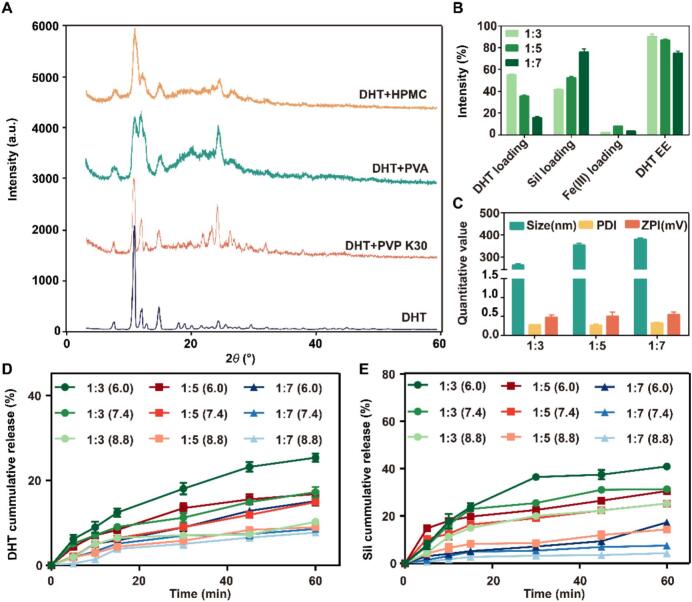
Preparation process investigation of DHT NC and DHT NC@SF MPN. (A) X-ray diffraction patterns of DHT NC prepared with different dispersants. (B) Drug loading and encapsulation efficiency of DHT NC@SF MPN at different dosage ratios. (C) Particle size, PDI and zeta potential of DHT NC@SF MPN at different dosage ratios. (D) DHT release at different pH conditions from DHT NC@SF MPN preparing from different ratios of Sil to Fe(III). (E) Sil release at different pH conditions from DHT NC@SF MPN preparing from different ratios of Sil to Fe(III). All data were shown as mean ± SD, *n* = 6.

In this investigation, the DHT NC@SF MPN was designed with a core-shell structure, which was composed of nanocrystal and polyphenol-metal network. Firstly, the DHT NC was prepared by the solvent/anti-solvent precipitation method, and the X-ray diffractometer was used to evaluate the influence of dispersants type on the crystalline form. As shown in the XRD spectra (Fig. 2A), the DHT NC with PVP K30 as dispersant showed flat baseline and sharp diffraction peak, indicating it had a high degree of crystallinity. However, when using PVA or HPMC as dispersant, the baseline slowly drifts and the diffraction peaks widened and split, indicating the crystal lattice was unstable and flawed. Then, an orthogonal test was used to optimize the preparation process of DHT NC. As shown in Table 1, the particle size was the smallest and the PDI was the lowest at the A3B1C1D3 condition (ratio of dispersants to DHT, 1:5; stirring speed, 30000 rpm; homogenization pressure, 10000 spi; cycle times, 60).

**Table 1:** Orthogonal experiments results of DHT NC preparation.

Experiment Number	Factors				Indicators	
	A: PVP K30 dosage	B: Stirring speed (rmp)	C: Pressure (spi)	D:Cycle number (times)	Particle size (nm)	PDI
1	1:1	3000	10,000	20	285.9 ± 17.9	0.413 ± 0.111
2	1:1	4000	15,000	40	394.1 ± 14.7	0.491 ± 0.182
3	1:1	5000	20,000	60	394.1 ± 14.7	0.491 ± 0.182
4	1:3	3000	20,000	40	304.0 ± 3.6	0.380 ± 0.026
5	1:3	4000	10,000	60	192.7 ± 10.0	0.283 ± 0.032
6	1:3	5000	15,000	20	510.1 ± 18.8	0.718 ± 0.043
7	1:5	3000	15,000	60	193.9 ± 4.5	0.255 ± 0.039
8	1:5	4000	20,000	20	358.7 ± 10.3	0.670 ± 0.024
9	1:5	5000	15,000	40	237.0 ± 2.8	0.358 ± 0062
order of factors					D>C>B>A	D>C>B>A
optimizing combination					A3B1C1D3	A1B1C1D3
unified preparation process					A3B1C1D3	

All data were shown as mean ± SD.

Subsequently, Sil was coordinated with Fe(III) to synthesize polyphenol-metal network, which was coated on the DHT NC to form the DHT NC@SF MPN. With the molar ratio of Sil and Fe(III) increasing from 1:3 to 1:7, the Sil content increased from 41.88 % to 78.93 %, while the DHT content decreased from 56.08 % to 16.08 %. And the DHT encapsulation efficiency decreased from 55.28 % to 15.75 % (Fig. 2B). In addition, the particle size of the DHT NC@SF MPN was the smallest (262.12 ± 5.61 nm) at molar ratio of 1:3 (Fig. 2C). More importantly, the SF MPN disassembled more quickly at molar ratio of 1:3 than other molar ratios (Fig. 2D&2E). Therefore, the molar ratio of 1:3 (Sil/Fe(III)) was adopted for preparing the SF MPN.

### 3.2 DHT NC@SF MPN characterization

**Fig. 3:**
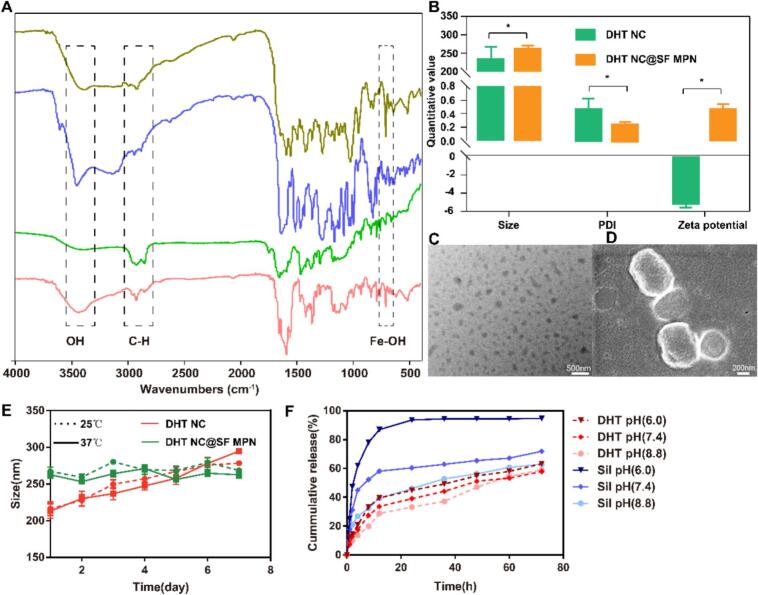
Characterization of the DHT NC@SF MPN. (A) IR spectra of DHT, Sil, DHT NC and DHT NC@SF MPN. (B) Particle size, PDI and zeta potential of DHT NC and DHT NC@SF MPN. (C) TEM image of DHT NC@SF MPN. (D) SEM image of DHT NC@SF MPN. (E) Particle size variation of DHT NC and DHT NC@SF MPN over 7 days. (F) Cumulative drug release from the DHT NC@SF MPN at different pH. All data were shown as mean ± SD, *n* = 6.

Infrared spectroscopy was used to confirm the structure of DHT NC@SF MPN. As shown in Fig. 3A, a new peak clearly appeared at 680 cm^−1^, which was characteristic peak of Fe(III)-hydroxy complex. It proved that the hydroxy of Sil had chelated with Fe(III). And the DHT NC@SF MPN exhibited a stronger hydroxide (OH) peak at 3439 cm^−1^ compared with DHT NC, and the hydrogen carbon (CH) peak intensity at 2920 cm^−1^ decreased after coating with SF MPN. The DHT NC displayed spherical or ellipsoidal shape with particle size approximately 224 nm (Fig. 3B). After coating with SF MPN, the particle size increased to 262 nm. The DHT NC@SF MPN displayed approximately spherical shape with core-shell structure (Fig. 3C&3D). When the DHT NC was placed at 25 °C or 37 °C for 1 week, the particle size increased from 173.12 ± 8.13 nm to 278.40 ± 3.04 nm, and the PDI increased from 0.28 ± 0.01 to 0.32 ± 0.04. While, there was no obvious change in particle size and PDI for the DHT NC@SF MPN, which demonstrated that coating with SF MPN could improve the thermodynamically unstable property of DHT NC (Fig. 3E). Furthermore, the drug release of the DHT NC@SF MPN was investigated under different physiological pH environment (Fig. 3F). The DHT NC@SF MPN released faster at pH 6.0 condition than at pH 7.4 or pH 8.8 condition, which might be due to that the SF MPN coating degraded faster in acidic environment than in the alkaline environment. As reported, the polyphenol-metal coordinate was easily dissociated after protonation of the phenolic hydroxyl groups, so the SF MPN possessed acidic responsive ability (Zhao et al., 2018). Therefore, it could be inferred that the DHT NC@SF MPN could avoid drug leakage during blood circulation and responsively dissociate under the acidic tumor micro-environment to release both DHT and Sil for efficient tumor therapy.

### 3.3 Pharmacodynamics on tumor cells

**Fig. 4:**
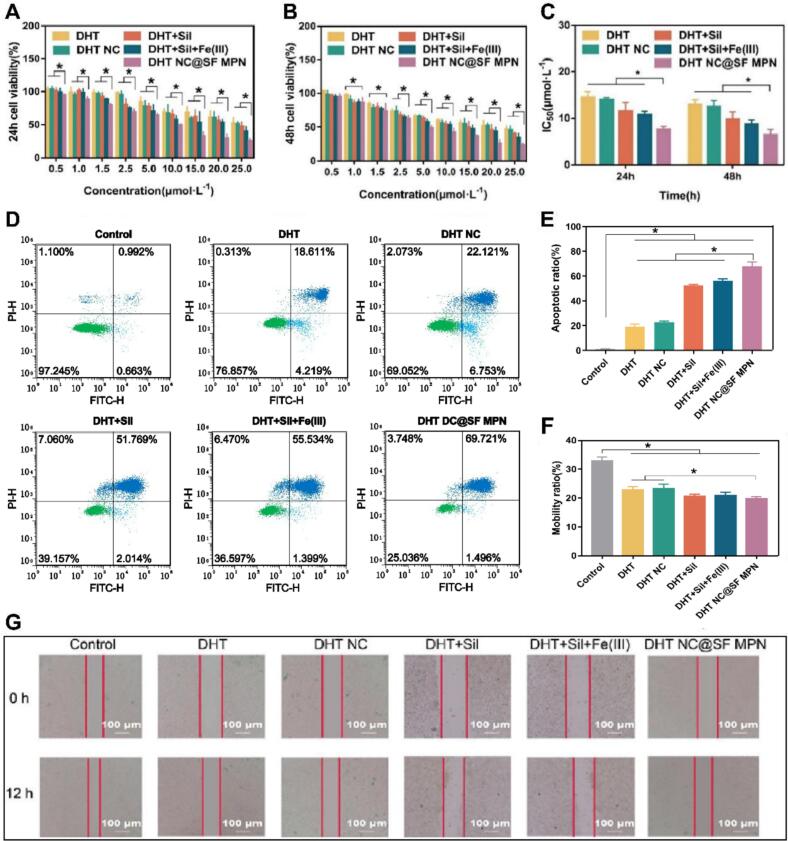

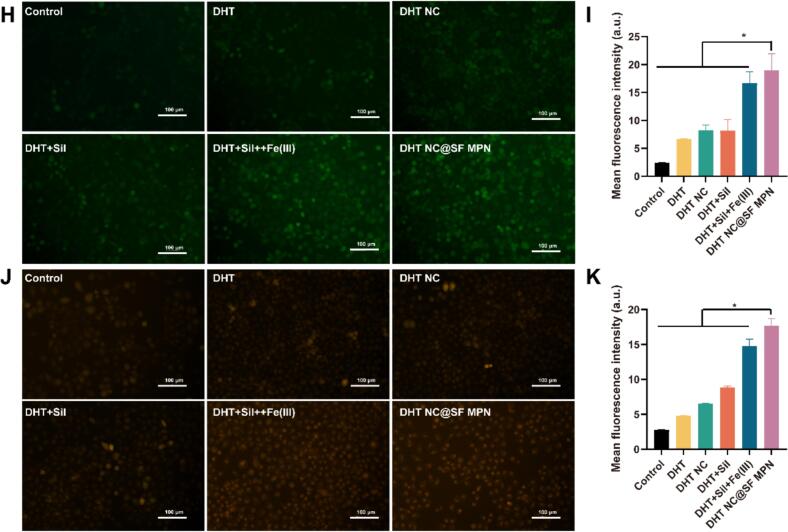
The pharmacodynamics of DHT NC@SF MPN on HGC 27 cells. (A) Viability of HGC 27 cells treated with different drug groups at different concentrations for 24 h. (B) Viability of HGC 27 cells treated with different groups at different concentrations for 48 h. (C) IC_50_ of different drug administration groups. (D) Images of cell apoptosis determined by flow cytrometry following treated with different drugs. (E) Apoptosis rate of different drug administration groups. (F) Cell migration rate of different drug administration groups (*n* = 3). (G) Images of scratch area after treating with different drugs at 0 h and 12 h. (H) Confocal images of HGC-27 cells after staining with DCFH-DA. (I) Quantitative analysis of intracellular ROS levels. (J) Confocal images of HGC-27 cells after staining with FerroOrange. (K) Quantitative analysis of intracellular Fe^2+^ levels. All data were shown as mean ± SD (*n* = 6), *p < 0.05.

The *in vitro* anti-tumor pharmacodynamics of the DHT NC@SF MPN was comprehensively evaluated on HGC 27 cells. Firstly, all drug administration groups exhibited certain dose-effect relationship, the cell survival rate of all groups showed a downward trend with the increase of drug concentration (Fig. 4A&4B). Among them, the DHT NC@SF MPN group showed stronger cytotoxicity than other groups, the IC_50_ of the DHT NC@SF MPN group (7.60 μmol·L^−1^) was almost half of the DHT (14.47 μmol·L^−1^) and DHT NC (13.96 μmol·L^−1^) groups, and far less than the DHT + Sil (11.48 μmol·L^−1^), DHT + Sil + Fe(III) (10.72 μmol·L^−1^) groups (Fig. 4C).

Subsequently, the potential of inducing cell apoptosis was evaluated using flow cytometry. Compared with the control group, drug administration groups increased cell apoptosis rate to varying degrees (*p* < 0.05) (Fig. 4D). And the cell apoptosis rate of the DHT NC@SF MPN group (65.00 %) was significantly higher than that of other four administration groups (DHT 20.91 %, DHT NC 23.75 %, DHT + Sil 53.56 %, DHT + Sil + Fe(III) 57.39 %) (*p* < 0.05), indicating that the DHT NC@SF MPN also could promote the cell apoptosis inducing ability of the DHT and Sil (Fig. 4E). Besides, the cell migration inhibition ability of the DHT NC@SF MPN was finally examined by a cell scratch experiment. For each drug administration group, their area of scratch was obvious larger than the control group (Fig. 4F&4G), and the migration rate of the DHT NC@SF MPN group (19.75 %) was significantly lower than the DHT (22.85 %) and DHT NC (23.27 %) groups (*p* < 0.05). These results demonstrated the DHT NC@SF MPN could efficiently inhibit tumor cell proliferation, migration and enhance cell apoptosis.

Finally, cell ferroptosis induced by the DHT NC@SF MPN nanocomplex was evaluated by determining levels of redox cytokines. As shown in Fig. 4H-4K, treatment with the DHT NC@SF MPN nanocomplex resulted in a significant increase in ROS and Fe^2+^ level in HGC-27 cells. It could be inferred that intracellular Fe^3+^ could react with GSH to produce Fe^2+^, which further converted H_2_O_2_ into highly reactive hydroxyl radicals (^●^OH). These radicals initiated a chain reaction of oxidative damage, culminating in elevated reactive oxygen species (ROS) levels and extensive oxidative stress.

### 3.4 Cellular uptake and intracellular distribution

**Fig. 5:**
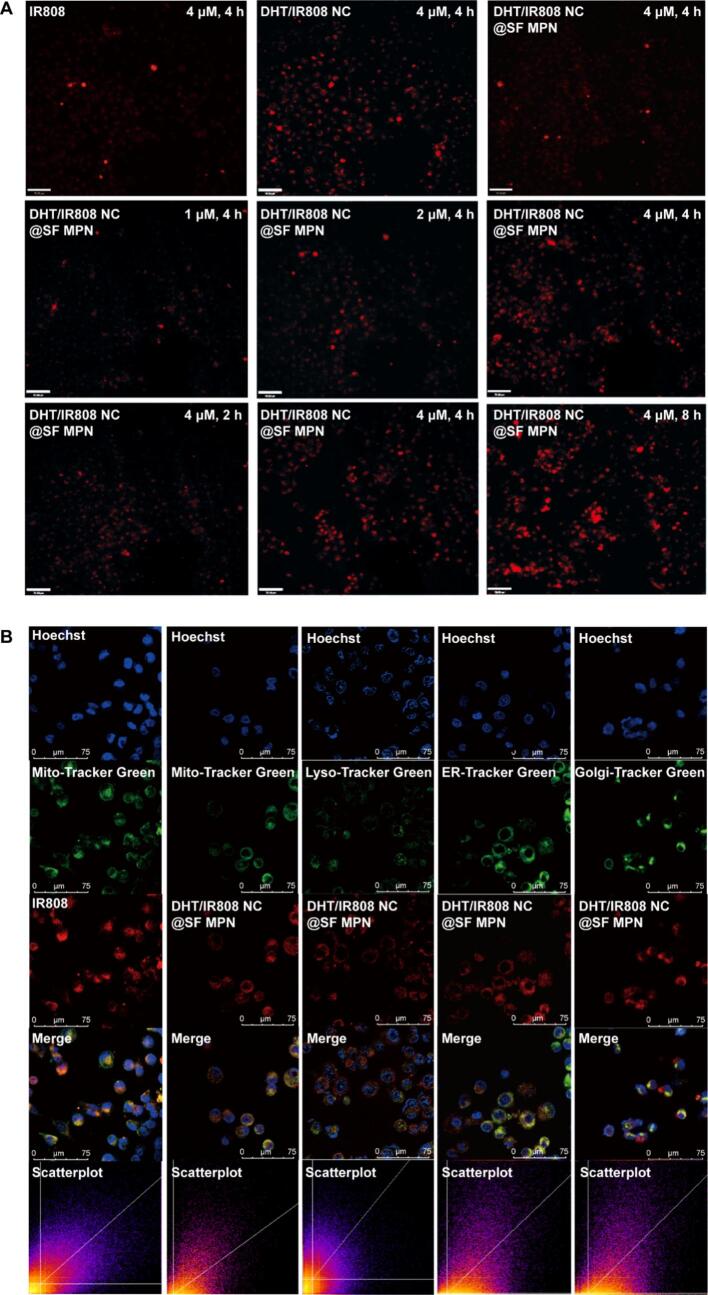

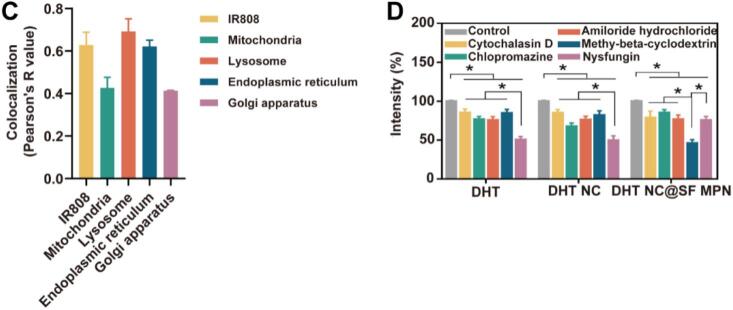
HGC 27 cells uptake, intracellular distribution and uptake pathway of DHT NC@SF MPN. (A) Laser confocal images of DHT/IR808 NC and DHT/IR808 NC@SF MPN taken at different concentrations with different incubation times. Scale bar = 70 μm. (B) Colocalized laser-confocal images of the DHT/IR808 NC@SF MPN with the nucleus, mitochondria, lysosomes, the endoplasmic reticulum, and the Golgi apparatus. Scale bar = 75 μm. (C) Quantification analysis by Pearson's correlation coefficient (PCC). (D) Intracellular DHT content of HGC 27 cells after treatment with different protein inhibitors (*n* = 6). All date were shown as mean ± SD, *p < 0.05.

The DHT NC and DHT NC@SF MPN were doped with red fluorescent substance IR808 to explore their intracellular distribution. As shown in Fig. 5A, the fluorescence intensity of the DHT NC and DHT/IR808 NC@SF MPN was stronger than the free IR808, indicating nano-sized particle was beneficial for cellular uptake. The fluorescence intensity of the DHT/IR808 NC@SF MPN group was gradually augmented with the incubating time and incubating drug concentration, demonstrating its cellular uptake was time/concentration-dependent. Further, major organelles (nucleus, mitochondria, lysosome, endoplasmic reticulum and Golgi apparatus) were stained with specific fluorescent probes to localize the intracellular distribution of the DHT/IR808 NC@SF MPN. As shown in Fig. 5B, the red fluorescence of the DHT/IR808 NC@SF MPN was widely distributed in mitochondria, lysosomes and endoplasmic reticulum. Co-localization analysis (Fig. 5C) suggested the nanocomplex mainly distributed in lysosomes (Pearson's R value>0.5), which was in accordance with that nanoparticles uptake into cells would firstly enter lysosomes for degradation and metabolism. And the distribution in endoplasmic reticulum demonstrated that the DHT/IR808 NC@SF MPN could achieve lysosomal escape.

Different endocytic protein inhibitors were employed to further explore the pathways of cellular uptake of DHT NC@SF MPN. As shown in Fig. 5D, compared with the control group, the intracellular DHT content was significantly decreased after treatment with inhibitors (*p* < 0.05), which suggested that the DHT NC and DHT NC@SF MPN entry into HGC 27 cells through multiple pathways. After inhibiting the caveolin mediated pathway with nystatin, the contents of DHT NC taken up by cells were only approximately 50 % of the control group, indicating that the caveolin mediated pathway was the main pathway for the DHT NC cellular uptake. While, for the DHT NC@SF MPN group, the content of DHT in cells significantly decreased after treatment with methyl β cyclodextrin, which mainly inhibited the lipid raft/caveolin mediated pathway. The different transport pathways of DHT NC and DHT NC@SF MPN might be explained by their different particle size and surface properties.

### 3.5 Pharmacodynamics on tumor-bearing mice

**Fig. 6:**
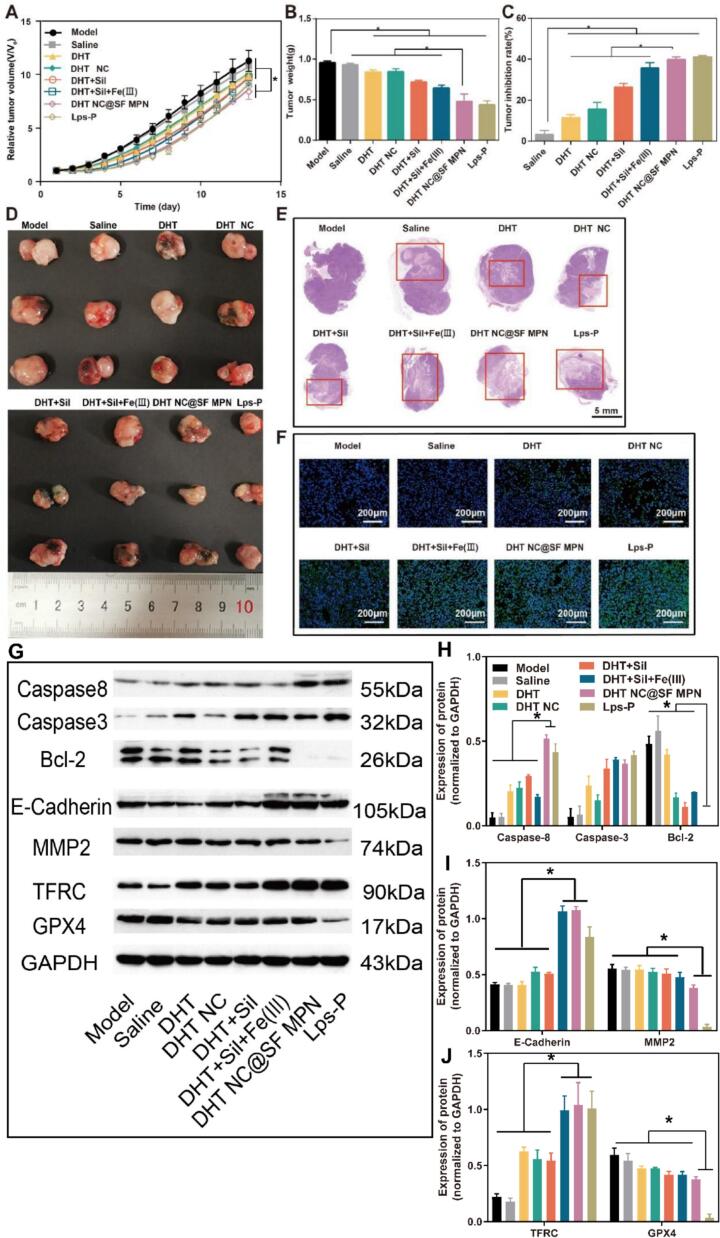
The *in vivo* pharmacodynamics and action mechanism of DHT NC@SF MPN on HGC 27 tumor-bearing nude mice. (A) The relative tumor volume and (B) corresponding tumor weights of tumor-bearing mice over time after 14 days of different treatment. (C)Tumor growth inhibition of different treatment groups. (D) Representative tumor photographs of mice at 14 days after different treatments. (E) H&E staining of tumor sections, scale bar = 5 mm. (F) TUNEL staining of tumor sections, scale bar = 200 μm. (G) Expression of apoptosis, metastasis and ferroptosis related proteins in tumor tissues analyzed by western blot. (H-J) Quantitative protein expression by relative gray values with GAPDH as reference. All data were shown as mean ± SD (*n* = 6), ^⁎^*p* < 0.05.

*In vivo* pharmacodynamics were evaluated on the HGC 27 tumor-bearing nude mice. Compared with the model and saline groups, tumor volume and tumor weight of each administration group were significantly reduced (*p* < 0.05) (Fig. 6A&6B). The tumor inhibition rate of the DHT NC@SF MPN group was the highest, and it exhibited equivalent antitumor efficiency compared with the positive control group (Lps-P) (*p* > 0.05) (Fig. 6C&6D). From the H&E staining of the tumor sections, it could be seen that a small amount of necrotic tumor tissues appeared in the center of the tumor body in the model group and the saline group (Fig. 6E). This phenomenon could be ascribed to the rapid growth of the tumor and delayed construction of blood vessels, which resulted in the lack of nutrients and oxygen in the central tumor and further caused tumor death (Leek et al., 1999). The necrotic area size of DHT NC@SF MPN and Lps-P groups significantly augmented compared with other drug administration groups, indicating they had better anti-tumor efficiency. TUNEL staining was also conducted for apoptosis analysis. As shown in the Fig. 6F, green fluorescence almost could not be observed in the model and saline groups, indicating there was no tumor cell apoptosis. The DHT NC@SF MPN and Lps-P groups had the largest green fluorescence area among all drug administration groups, indicating they could efficiently induce tumor cell apoptosis. Besides, when treatment with drug combination, the apoptosis rate was higher than each single drug. The expression of proteins involved in tumor cell apoptosis, metastasis and ferroptosis was also investigated (Fig. 6G-6J). Both DHT NC@SF MPN and Lps-P could significantly up-regulate caspase3 and caspase8 expression while down-regulate Bcl-2 expression, which were favorable to tumor cell apoptosis. Undoubtedly, when combination with Fe(III), the TFRC expression increased and the GPX4 expression decreased, which suggested the ferroptosis was activated. These results could demonstrate the antitumor effects of DHT NC@SF MPN was multi-pathway related. The DHT DC@SF MPN group exhibited a much weaker regulatory effect on MMP2 and GPX4 compared to the Lps-P group, which might be due to specific inhibition of paclitaxel on both proteins.

### 3.6 Safety evaluation

**Fig. 7:**
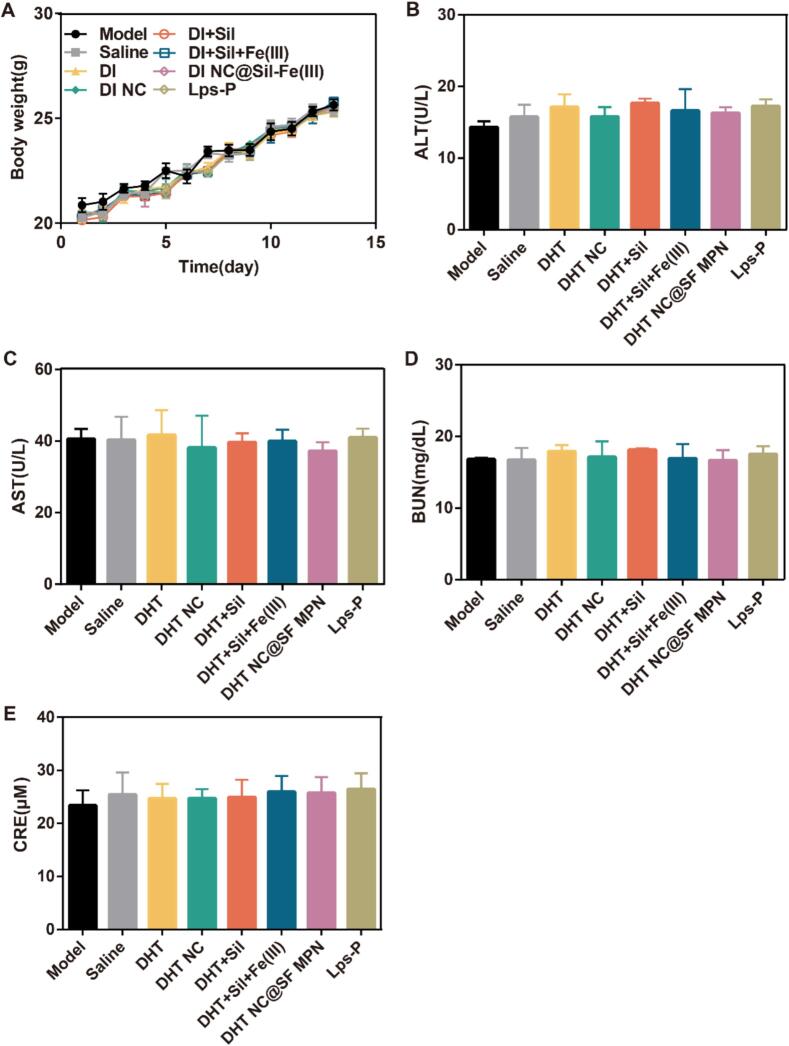

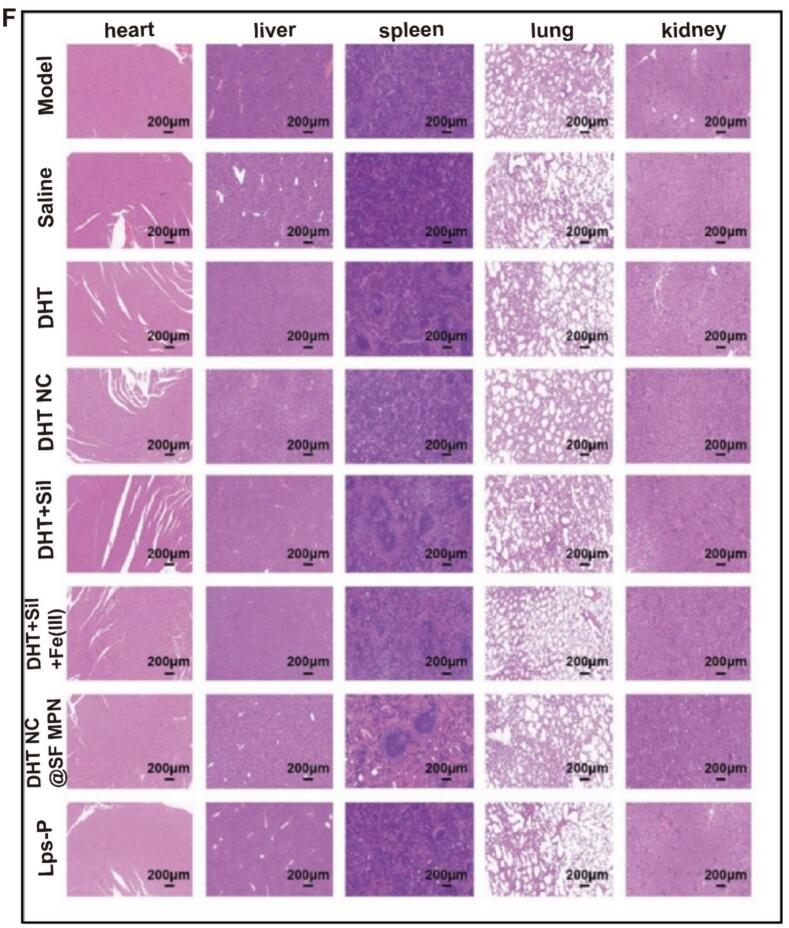
(A) Body weight changes during the establishment of mouse model and treatment period. (B-E) Serum levels of ALT, AST, BUN and CRE, (n = 6). (F) H&E staining of major tissue sections.

In the end, the potential toxicity was evaluated carefully. The weight change curves of mice in each group were similar to those in the normal group, and there was no significant body weight loss in the treatment groups, demonstrating that the systemic toxicity was negligible (Fig. 7A). The biochemical examination revealed that the levels of ALT, AST, CRE, and BUN between the control group and each treatment group did not change significantly (*p* > 0.05) (Fig. 7B-7E). The H&E staining images of normal tissue sections showed no evident tissue damage or tumor metastasis foci (Fig. 7F). The superior safety probably due to the intra-tumoral administration method, which efficiently avoided the whole body distribution of drugs.

## 4 Conclusions

In summary, we constructed a carrier-free type nanoplatform integrating metal-phenolic network and nanocrystal, which was a facile strategy for simultaneously delivering multi-drugs. Compared with conventional nanomedicine, the specific advantages of the DHT NC@SF MPN nanocomplex included high drug loading, facile preparation process, and distinctive mechanism of action. The nanocomplex was almost consisted of three therapeutic ingredients, its drug loading was nearly 100 %. Whereas, the drug loading of conventional nanomedicine such as liposome, nanoparticle, dendrimer, *etc.*, commonly less than 20 %. Without use of nanomaterials made the preparation process simple and economical. Moreover, it avoided nanomaterial toxicity, which was beneficial for clinical translation. Due to the complexity of tumor, it is a challenge to obtain satisfactory therapeutic effects through a single chemotherapeutic drug or therapy modality. The nanocomplex could co-deliver three chemotherapeutic drugs and produce synergistic therapeutic outcomes. It should be noted that when integrating these three drugs into an entity its treatment efficiency far exceeded that of simple addition. Despite the promising results, it was necessary to remind the limitations in application, for example not all drugs and metal ions were suitable for constructing metal-phenolic network. Moreover, anti-tumor efficacy with different administration routs, like oral administration and intravenous administration, might be needed to be further assessed.

## CRediT authorship contribution statement

**Pengkai Ma:** Writing – review & editing, Writing – original draft, Supervision, Funding acquisition, Conceptualization. **Ziqi Jing:** Methodology, Formal analysis, Data curation. **Xue Wang:** Methodology, Formal analysis. **Xiaoya Liu:** Writing – original draft. **Zirui Tan:** Writing – original draft. **Yujie Zhang:** Resources, Project administration. **Zhijun Wang:** Resources, Project administration, Funding acquisition.

## Ethical approval statement

Animal research was conducted by the Guide for the Care and Use of Laboratory Animals (China). The animal study protocol was approved by the Animal Care and Use Committee of Beijing University of Chinese Medicine (the Laboratory Animal Use Permit Number: BUCM-4-20,220,627-2126).

## Declaration of competing interest

The authors declare the following financial interests/personal relationships which may be considered as potential competing interests:(Pengkai Ma reports financial support was provided by National Natural Science Foundation of China. Zhijun Wang reports financial support was provided by National Natural Science Foundation of China. Pengkai Ma reports financial support was provided by Fundamental Research Funds for the Central Universities. If there are other authors, they declare that they have no known competing financial interests or personal relationships that could have appeared to influence the work reported in this paper.)

## Data Availability

Data will be made available on request.

## Appendix A Supplementary data

Supplementary material Image 1
