# Supplementary material for: Carrier-free multi-components self-delivery nanocomplex for tumor synergistic therapy
Source: Int J Pharm X. 2025 Nov 12;10:100443. doi: 10.1016/j.ijpx.2025.100443 (PMC12664389; doi:10.1016/j.ijpx.2025.100443)
Supplement: Supplementary file 1 — Supplementary material [file mmc1.docx]

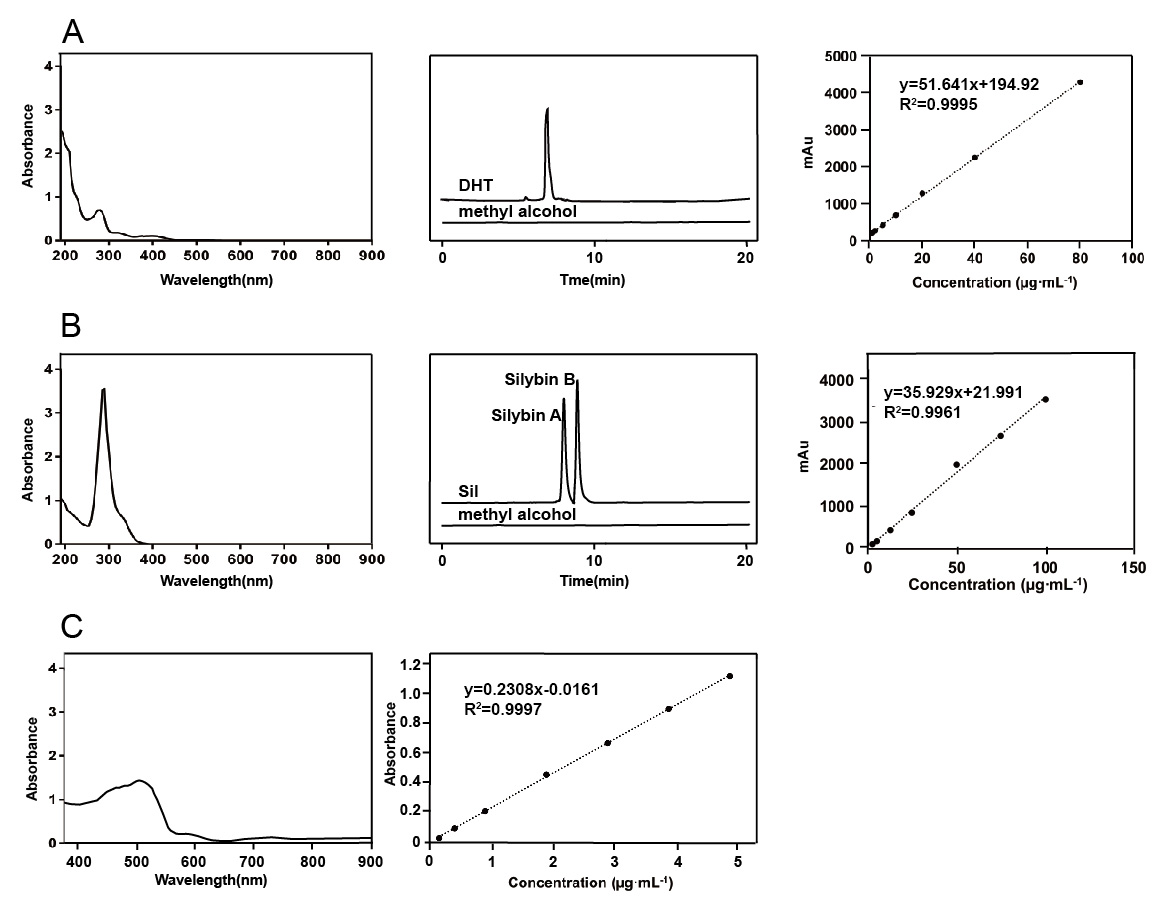


Supplementary Fig.S1 (A) and (B) UV-Vis scanning spectrum, chromatogram and standard curve of DHT and Sil. (C) UV-Vis scanning spectrum and standard curve of o-Phenanthroline-Fe(III) complex.
